# Supplementary material for: Conjugates for use in peptide therapeutics: A systematic review and meta-analysis
Source: PLoS One. 2022 Mar 8;17(3):e0255753. doi: 10.1371/journal.pone.0255753 (PMC8903268; doi:10.1371/journal.pone.0255753)
Supplement: S1 Table — (PDF) [file pone.0255753.s002.pdf]

Table S1: Systematic Search Strategy used for PubMed, Scopus, and SciFinder

Search strategy for PubMed	
**Search #1**	**Therapeutic Peptides Keywords**
**Search query**	"peptide-based therap*"[tiab] OR "peptide therap*"[tiab] OR "peptide drug*"[tiab] OR "therapeutic peptide*"[tiab] OR "anticancer peptide*"[tiab]
**Search #2**	**Therapeutic Peptides MeSH terms**
**Search query**	"Peptides"[Mesh] OR "Biological Products"[Mesh]
**Search #3**	**Conjugates keywords**
**Search query**	"peptide conjugate*"[tiab] OR "PEG"[tiab] OR "polyethylene glycol*"[tiab] OR "liposome*"[tiab] OR "nanoparticle*"[tiab] OR "dextran"[tiab] OR "unnatural amino acid*"[tiab] OR "PASylation"[tiab] OR "HESylation"[tiab] OR "HA conjugation"[tiab] OR "HAylation"[tiab] OR "XTEN"[tiab] OR "PEGylation"[tiab] OR "ELPylation"[tiab] OR "Elastin-like polypeptide*"[tiab] OR "N-glycosylation"[tiab] OR "Polysialylation"[tiab] OR "conjugate*"[tiab]
**Search #4**	**Conjugates MeSH terms**
**Search query**	"Drug Carriers"[Mesh] OR "Polyethylene Glycols"[Mesh] OR "Liposomes"[Mesh] OR "Nanoparticles"[Mesh] OR "Lipids"[Mesh] OR "Hydroxyethyl Starch Derivatives"[Mesh] OR "Hyaluronic Acid"[Mesh] OR "Glycosylation"[Mesh] OR "Sialic Acids"[Mesh] OR "Serum Albumin"[Mesh]
**Search #5**	**Half-life keywords**
**Search query**	"half-life"[tiab] OR "half life"[tiab]
**Search #6**	**Half-life MeSH terms**
**Search query**	"Half-Life"[Mesh]
**Search #7**	**#1 OR #2**
**Search query**	("peptide-based therap*"[tiab] OR "peptide therap*"[tiab] OR "peptide drug*"[tiab] OR "therapeutic peptide*"[tiab] OR "anticancer peptide*"[tiab]) OR ("Peptides"[Mesh] OR "Biological Products"[Mesh])
**Search #8**	**#3 OR #4**
**Search query**	("peptide conjugate*"[tiab] OR "PEG"[tiab] OR "polyethylene glycol*"[tiab] OR "liposome*"[tiab] OR "nanoparticle*"[tiab] OR "dextran"[tiab] OR "unnatural amino acid*"[tiab] OR "PASylation"[tiab] OR "HESylation"[tiab] OR "HA conjugation"[tiab] OR "HAylation"[tiab] OR "XTEN"[tiab] OR "PEGylation"[tiab] OR "ELPylation"[tiab] OR "Elastin-like polypeptide*"[tiab] OR "N-glycosylation"[tiab] OR "Polysialylation"[tiab] OR "conjugate*"[tiab]) OR ("Drug Carriers"[Mesh] OR "Polyethylene Glycols"[Mesh] OR "Liposomes"[Mesh] OR "Nanoparticles"[Mesh] OR "Lipids"[Mesh] OR "Hydroxyethyl Starch Derivatives"[Mesh] OR "Hyaluronic Acid"[Mesh] OR "Glycosylation"[Mesh] OR "Sialic Acids"[Mesh] OR "Serum Albumin"[Mesh])
**Search #9**	**#5 OR #6**
**Search query**	("half-life"[tiab] OR "half life"[tiab]) OR ("Half-Life"[Mesh])
**Search #10**	**#7 AND #8 AND #9**
**Search query**	((("peptide-based therap*"[tiab] OR "peptide therap*"[tiab] OR "peptide drug*"[tiab] OR "therapeutic peptide*"[tiab] OR "anticancer peptide*"[tiab]) OR ("Peptides"[Mesh] OR "Biological Products"[Mesh])) AND (("peptide conjugate*"[tiab] OR "PEG"[tiab] OR "polyethylene glycol*"[tiab] OR "liposome*"[tiab] OR "nanoparticle*"[tiab] OR "dextran"[tiab] OR "unnatural amino acid*"[tiab] OR "PASylation"[tiab] OR "HESylation"[tiab] OR "HA conjugation"[tiab] OR "HAylation"[tiab] OR "XTEN"[tiab] OR "PEGylation"[tiab] OR "ELPylation"[tiab] OR "Elastin-like polypeptide*"[tiab] OR "N-glycosylation"[tiab] OR "Polysialylation"[tiab] OR "conjugate*"[tiab]) OR ("Drug Carriers"[Mesh] OR "Polyethylene Glycols"[Mesh] OR "Liposomes"[Mesh] OR "Nanoparticles"[Mesh] OR "Lipids"[Mesh] OR "Hydroxyethyl Starch Derivatives"[Mesh] OR "Hyaluronic Acid"[Mesh] OR "Glycosylation"[Mesh] OR "Sialic Acids"[Mesh] OR "Serum Albumin"[Mesh]))) AND (("half-life"[tiab] OR "half life"[tiab]) OR ("Half-Life"[Mesh]))
**Search #11**	**NOT string**
**Search query**	"vaccine*"[tiab] OR "Vaccination"[tiab] OR "Vaccines"[MeSH Terms] OR "Vaccination"[MeSH Terms] OR "Antibodies"
**Search #12**	**#7 AND #8 AND #9 NOT #11**
**Search query**	(((("peptide-based therap*"[tiab] OR "peptide therap*"[tiab] OR "peptide drug*"[tiab] OR "therapeutic peptide*"[tiab] OR "anticancer peptide*"[tiab]) OR ("Peptides"[Mesh] OR "Biological Products"[Mesh])) AND (("peptide conjugate*"[tiab] OR "PEG"[tiab] OR "polyethylene glycol*"[tiab] OR "liposome*"[tiab] OR "nanoparticle*"[tiab] OR "dextran"[tiab] OR "unnatural amino acid*"[tiab] OR "PASylation"[tiab] OR "HESylation"[tiab] OR "HA conjugation"[tiab] OR "HAylation"[tiab] OR "XTEN"[tiab] OR "PEGylation"[tiab] OR "ELPylation"[tiab] OR "Elastin-like polypeptide*"[tiab] OR "N-glycosylation"[tiab] OR "Polysialylation"[tiab] OR "conjugate*"[tiab]) OR ("Drug Carriers"[Mesh] OR "Polyethylene Glycols"[Mesh] OR "Liposomes"[Mesh] OR "Nanoparticles"[Mesh] OR "Lipids"[Mesh] OR "Hydroxyethyl Starch Derivatives"[Mesh] OR "Hyaluronic Acid"[Mesh] OR "Glycosylation"[Mesh] OR "Sialic Acids"[Mesh] OR "Serum Albumin"[Mesh]))) AND (("half-life"[tiab] OR "half life"[tiab]) OR ("Half-Life"[Mesh]))) NOT ("vaccine*"[tiab] OR "Vaccination"[tiab] OR "Vaccines"[MeSH Terms] OR "Vaccination"[MeSH Terms] OR "Antibodies"[MeSH Terms]) AND ((fft[Filter]) AND (english[Filter]))

Table S1 continued

**Search strategy for Scopus**	
**Search #1**	**Therapeutic Peptides Keywords**
**Search query**	TITLE-ABS-KEY ("peptide-based therap*" OR "peptide therap*" OR "peptide drug*" OR "therapeutic peptide*" OR "anticancer peptide*")
**Search #2**	**Conjugates keywords**
**Search query**	TITLE-ABS-KEY ("peptide conjugate*" OR "PEG" OR "polyethylene glycol*" OR "liposome*" OR "nanoparticle*" OR "dextran" OR "unnatural amino acid*" OR "PASylation" OR "HESylation" OR "HA conjugation" OR "HAylation" OR "XTEN" OR "PEGylation" OR "ELPylation" OR "Elastin-like Polypeptide" OR "N-glycosylation" OR "Polysialylation" OR "conjugate")
**Search #3**	**Half-life keywords**
**Search query**	TITLE-ABS-KEY("half-life" OR "half life")
**Search #4**	**NOT string**
**Search query**	TITLE-ABS-KEY (“vaccine*” OR "Vaccination" OR "Vaccines" OR "Antibodies")
**Search #5**	**#1 AND #2 AND #3 AND NOT #5**
**Search query**	(TITLE-ABS-KEY("peptide-based therap*" OR "peptide therap*" OR "peptide drug*" OR "therapeutic peptide*" OR "anticancer peptide*")) AND (TITLE-ABS-KEY("peptide conjugate*" OR "PEG" OR "polyethylene glycol*" OR "liposome*" OR "nanoparticle*" OR "dextran" OR "unnatural amino acid*" OR "PASylation" OR "HESylation" OR "HA conjugation" OR "HAylation" OR "XTEN" OR "PEGylation" OR "ELPylation" OR "Elastin-like Polypeptide" OR "N-glycosylation" OR "Polysialylation" OR "conjugate")) AND (TITLE-ABS-KEY("half-life" OR "half life")) AND NOT (TITLE-ABS-KEY(“vaccine*” OR "Vaccination” OR "Vaccines” OR "Antibodies")) AND (LIMIT-TO (PUBYEAR, 2020) OR LIMIT-TO (PUBYEAR, 2019) OR LIMIT-TO (PUBYEAR, 2018) OR LIMIT-TO (PUBYEAR, 2017) OR LIMIT-TO (PUBYEAR, 2016) OR LIMIT-TO (PUBYEAR, 2015)) AND (LIMIT-TO (LANGUAGE, "English"))
**Search strategy for SciFinder**	
**Search #1**	Research Topic "peptide therapeutic"
**Limiters**	Publication Years: 2015 - 2020<br>Languages: English
**Search #2**	Research Topic "peptide conjugate"
**Limiters**	Publication Years: 2015 - 2020<br>Languages: English
**Search #3**	Research Topic "half-life"
**Limiters**	Publication Years: 2015 - 2020<br>Languages: English
**Search #4**	Intersect answer sets for searches #1, #2 and #3 (#1 ∩ #2 ∩ #3)
**Limiters**	Publication Years: 2015 - 2020<br>Languages: English
**Search #5**	Research Topic "peptide therapeutic half-life and conjugate"
**Limiters**	Publication Years: 2015 - 2020<br>Languages: English
**Search #6**	Combine answer sets for searches #4 and #5 (#4 ∪ #5)
**Limiters**	Publication Years: 2015 - 2020<br>Languages: English
