# Supplementary material for: Conjugates for use in peptide therapeutics: A systematic review and meta-analysis
Source: PLoS One. 2022 Mar 8;17(3):e0255753. doi: 10.1371/journal.pone.0255753 (PMC8903268; doi:10.1371/journal.pone.0255753)
Supplement: S2 Table — (PDF) [file pone.0255753.s003.pdf]

Table S2: Characteristics of relevant studies captured in the systematic search.

Study ID	Peptide (Size)	Animal Model	Outcome Analysis	Conjugate	Conjugated Peptide	Half-life (h)
Bak 2020 (33)	GLP-1[N] 31mer (~3.41 kDa)	Female BALB/L mice	ELISA	HSA (Human Serum Albumin)	GLP1_16HSA	8.4
					GLP1_19HSA	7.4
					GLP1_28HSA	8
Chen 2016 (44)	Exendin-4[N] 39mer (~4.29 kDa)	Female BALB/C mice	Dynamic Positron Emission Tomography (PET) scanning	tEB (Evan's Blue dye derivative)	tEB-Exendin-4	0.34
Fawaz 2020 (38)	22A[A] (apo A1 mimetic peptide) 2.62 kDa*	Male SD rats	LC-MS	1-palmitoyl-2-oleoyl-sn-glycero-3-phosphocholine (POPC)	22A- POPC	3.3
				1,2-dimyristoyl-sn-glycero-3-phosphocholine (DMPC)	22A-DMPC	3
				1,2-dipalmitoyl-sn-glycero-3-phosphocholine (DPPC)	22A-DPPC	3.3
				1,2- distearoyl-sn-glycero-3-phosphocholine (DSPC)	22A-DSPC	3.3
Fu 2020 (45)	LEU[A] (leuroprolide) 1.21 kDa	Male SD rats	Ultra-Performance Liquid Chromatography tandem MS (UPLC-MS/MS)	PEG2K	PEG2K-LEU	0.53 ± 0.032
				PEG5K	PEG5K-LEU	1.28 ± 0.64
Fukushima 2019 (35)	Insulin[N] 5.8 kDa	Male ICR mice	Sandwich ELISA	Chondroitin (CH)	CH-C3-GlyA1-insulin	5.6
					CH-C3-LysB29-insulin	3.4
					CH-C3-GlyA1/LysB29-insulin	7.5
					CH-C6-GlyA1-insulin	5.6
					CH-C6-LysB29-insulin	2.2
					CH-C6-GlyA1/LysB29-insulin	9.4
					CH-C11-GlyA1-insulin	4.8
					CH-C11-LysB29-insulin	4.9
					CH-C11-GlyA1/LysB29-insulin	14

Table S2 continued

Fukushima 2019 continued				Heparosan (HPN)	HPN-C3-GlyA1-insulin	7.3
					HPN-C3-LysB29-insulin	6.1
					HPN-C3-GlyA1/LysB29-insulin	16.9
					HPN-C11-GlyA1-insulin	5.6
					HPN-C11-LysB29-insulin	6.9
					HPN-C11-GlyA1/LysB29-insulin	12.9
		Male SD rats		CH	CH-C3-GlyA1-insulin	5.7
				HPN	HPN-C3-GlyA1-insulin	8
Ichikawa 2018 (35)	GLP-1C[A] 38mer (~4.18 kDa)	Male ICR mice	ELISA	CH	CH70-EDA-(LMDS)-GLP-1C	32.9
					CH90-EDA-(LMDS)-GLP-1C	25.3
				HPN	HPN50-EDA-(LMDS)-GLP-1C	33.6
Kim 2019 (32)	GLP-1[N] 30mer (~3.3 kDa)	Male BALB/C mice	ELISA	HSA specific repebody	repebody+GLP-1	10.7
			Radioisotope kinetics (2 phase model)			8.57
Knadler 2015 (36)	Insulin lispro[A] (BIL) 5.8 kDa	Male SD rats	ELISA	PEG	[14C]BIL	5
					[125I]BIL	20
Lear 2020 (46)	peptide tyrosine tyrosine[N] (PYY2) 3.63 kDa	Male SD rats	UPLC-MS/MS	PEG/Fatty acids (S11)	S11-PYY2	14.4
Liu 2015 (39)	Hirulog-1[A] (Hirudin mimetic peptide) 2.39 kDa	SD rats	Electrospray ionization tandem MS (ESI-MS/MS)	Stearic acid	Acylated modified Hirulog	3.54 ± 0.97
McVicar 2017 (47)	TP508[A] (508-530 of human prothrombin)	Male CD-1 mice	Fluorescence measurements	PEG5k	PEG5k-TP508	0.19
				PEG20k	PEG20k-Cys14-TP508	1.17
					PEG20k-TP508	1.55
				PEG30k	PEG30k-TP508	4.3
Pessi 2019 (50)	HR2 peptide[H] (610-633 of EBOV GP2-a fusion protein)	BALB/c mice	unknown	PEG4-chol	EBOV 5	0.9
				PEG12-chol	EBOV 6	5.85

Table S2 continued

Ranganath 2015 (37)	PN-2921[H] - a novel IL-6 binding peptide created from a functionally inert scaffold<br>2.99 kDa	Male ICR Mice	LC-MS/MS	PEG40k (branched)	PN 2921 (PEGylated at residue 29)	23
		Male SD Rats				36
		Male cynomolgous monkeys				59
Tan 2017 (48)	Thymopentin[N] (TP5)<br>0.68 kDa	Male Wistar rats	Calculated using the Winnoline 5.2.1 software based on a non-compartmental model.	Myristic acid	TP5-MA	1.75 ± 0.72
Tang 2017 (40)	22A[A] (apo A1 mimetic peptide)<br>2.62 kDa*	Male SD rats	LC/MS	sHDL: DPPC & POPC 1:1 ratio	22A-sHDL	6.27 ± 16.6
Zorzi 2017(41)	UK18[A] (an inhibitor of urokinase)<br>2.41 kDa	Female SD rats	HPLC analysis	Palmitoyl tag (palm) - EYEKpalmEYE	tagged-UK18	7.4 ± 0.2

Notes: [N] = native peptide, [A] = analogue, [H] = heterologous peptide
*Computed by ExPASy ProtParam using primary sequences provided intext (ProtParam was accessed via https://web.expasy.org/protparam/) (51)
