# Supplementary material for: Conjugates for use in peptide therapeutics: A systematic review and meta-analysis
Source: PLoS One. 2022 Mar 8;17(3):e0255753. doi: 10.1371/journal.pone.0255753 (PMC8903268; doi:10.1371/journal.pone.0255753)
Supplement: S3 Table — (PDF) [file pone.0255753.s004.pdf]

Table S3: Breakdown of the Risk of Bias analysis for individual studies.

Study	Sequence generation	Baseline characteristics	Allocation concealment	Random housing	Performance Bias - Anonymization	Random outcome assessment	Incomplete outcome data	Selective reporting	Other sources of bias
Bak 2020 (33)	Unsure	Low	Unsure	Unsure	Unsure	Unsure	Unsure	Low	High
Chen 2016 (44)	Unsure	Low	Unsure	Unsure	Unsure	Unsure	Unsure	Low	High
Fawaz 2020 (38)	Unsure	Low	Unsure	Unsure	Unsure	Unsure	Low	Low	Unsure
Fu 2020 (45)	Unsure	High	Unsure	Unsure	Unsure	Unsure	Unsure	Low	Low
Fukushima 2019 (34)	Unsure	Low	Unsure	Low	Unsure	Unsure	Low	Low	High
Ichikawa 2018 (35)	Unsure	Low	Unsure	Unsure	Unsure	Unsure	Unsure	Low	Unsure
Kim 2019 (32)	Unsure	Low	Unsure	Unsure	Unsure	Unsure	Unsure	Low	Unsure
Knadler 2015 (36)	Unsure	Low	Unsure	Unsure	Unsure	High	Unsure	Low	Unsure
Lear 2020 (46)	Unsure	Low	Unsure	Low	Unsure	Unsure	Unsure	Low	Unsure
Liu 2015 (39)	Unsure	Unsure	Unsure	Unsure	Unsure	Unsure	Low	Low	Unsure
McVicar 2017 (47)	Unsure	Low	Unsure	Unsure	Unsure	Unsure	Unsure	Low	Unsure
Pessi 2019 (50)	Unsure	Unsure	Unsure	Unsure	Unsure	Unsure	High	High	Unsure
Ranganath 2015 (37)	Unsure	Low	Unsure	Unsure	Unsure	Unsure	Unsure	Low	Unsure
Tan 2017 (48)	Unsure	Low	Unsure	Unsure	Unsure	Unsure	Low	Low	Unsure
Tang 2017 (40)	Unsure	Low	Unsure	Unsure	Unsure	Unsure	High	Low	Unsure
Zorzi 2017 (41)	Unsure	Unsure	Unsure	Unsure	Unsure	Unsure	Low	Low	Unsure
