# Supplementary material for: Conjugates for use in peptide therapeutics: A systematic review and meta-analysis
Source: PLoS One. 2022 Mar 8;17(3):e0255753. doi: 10.1371/journal.pone.0255753 (PMC8903268; doi:10.1371/journal.pone.0255753)
Supplement: S1 Protocol — (PDF) [file pone.0255753.s005.pdf]

Citation

Ashan Wijesinghe, Sarika Kumari, Valerie Booth. Systematic Review and Meta Analysis of Peptide Conjugates for use in Peptide Therapeutics. PROSPERO 2020 CRD42020222579 Available from: https://www.crd.york.ac.uk/prospero/display_record.php?ID=CRD42020222579

Review question

Within the last 5 years, which biologically inert therapeutic peptide conjugate has lead to the greatest peptide half-life in the bloodstream of animals?

Context and rationale

With much of the body's physiological functions being governed by peptides that behave as intrinsic signalling molecules, peptides make ideal agents for therapeutic interventions of natural pathways. Peptides are also readily metabolised and excreted which prevents toxic accumulation of drug by-products that typically cause drugs to fail clinical trials. Additionally, with their high specificity, peptides ensure that unfavourable off-target binding side effects are minimized while ensuring an amplified cascade of reactions. However, being a biomolecule, peptide therapeutics are subject to plasma clearance due to proteolysis and renal clearance. To address this issue, research suggests conjugating peptides to other moieties. Therefore, we are interested in determining which of these conjugates has had the most success in extending in vivo half-life of therapeutic peptides.

Searches

For this systematic review, we will only be considering published literature available on the PubMed, SciFinder and Scopus databases. To do so, valid search strategies will be developed combining key search terms and MeSH terms based on the PICO statement (the specific search terms and MeSH terms used is attached as pdf). The English language filter will be applied and results limited to papers published between 1st Sept 2015 to 1st Sept 2020

Study designs to be included

Inclusion criteria:

No restrictions

Exclusion criteria:

No restrictions

Human disease modelled

Half-life of peptide therapeutics in the bloodstream

Animals/population

Inclusion criteria:

Must be healthy animals.

Exclusion criteria:

Must not be diseased animals.

Intervention(s), exposure(s)

Inclusion criteria:

- Must be a therapeutic peptide

- Must be intravenously administered

- Must be no larger than insulin in size (5.8 kDa)

- Must consist of L-amino acids

- Must be a linear or cyclic peptide

- May have minor modifications (ex: N & C terminal capping)

Exclusion criteria:

- Must not be a conventional small molecule antibiotic

- Must not be non-therapeutic peptides

- Must not consist of D-amino acids

- Must not be a peptide with major modifications (as in the case of daptomycin)

Comparator(s)/control

Inclusion criteria:

- Must be a biologically inert conjugate

- Must be a non-specific conjugate

Exclusion criteria:

- Must not be a vaccine conjugate

Other selection criteria or limitations applied

Inclusion criteria:

- Must be an English language publication

- Must have been published between 1 Sept 2015 - 1 Sept 2020

Exclusion criteria:

- The publication must not be a case report, abstract, review, note, book chapter, patent or conference letter

- Must not be a non-English language publication

Outcome measure(s)

Inclusion criteria:

- Must have data on plasma half-life (in vivo)

Exclusion criteria:

N/A

Study selection and data extraction

Procedure for study selection

The screening will take place in two stages, an initial title/abstract screening followed by a full text screening. Two reviewers will independently screen the entries and the third will resolve any conflicts. This process will take place on Covidence.

Prioritise the exclusion criteria

1. Must not be non-therapeutic peptides

2. Must not be a vaccine conjugate
3. Must not be a conventional small molecule antibiotic

4. Must not consist of D-amino acids

5. Must not be a peptide with major modifications (as in the case of daptomycin)

6. Must not be a non-English language publication

7. The publication must not be a case report, abstract, review, note or conference letter

Methods for data extraction

Data will be extracted manually from the text of the studies and from respective figures/graphs and tables. The extracted data will be checked by at least 1 reviewer.

Data to be extracted: study design

Data to be extracted includes information on the controls, the number of experimental groups (animals or people) and biological replicates vs technical replicates.

Data to be extracted: animal model

The species, age, disease status of the animals will be extracted. Including sites from which blood was drawn for outcome for outcome comparisons.

Data to be extracted: intervention of interest

The type of peptide being administered, its size, its function and the dose being administered will be extracted.

Data to be extracted: primary outcome(s)

Half-life in the bloodstream.

Data to be extracted: secondary outcome(s)

N/A

Data to be extracted: other

None

Risk of bias and/or quality assessment

By use of SYRCLE’s risk of bias tool.
The Risk of Bias will be assessed as per the signalling questions provided by Hooijmans et al. (2014) in their paper titled "SYRCLE’s risk of bias tool for animal studies." Assessment will be done by two reviewers and disagreements resolved by the third..

Strategy for data synthesis

Planned approach

A meta analysis is performed on the outcomes measures followed up by subgroup analyses.

Effect measure

Standardized mean difference

Effect models

A random-effects model will be used for our meta-analysis, however, if our I^2 value is >50%, we will consider adding a fixed-effects model analysis.

Heterogeneity

Heterogeneity will be assessed using the I^2 model.

Other

None

Analysis of subgroups or subsets

Subgroup analyses

We plan on analysis the difference in outcome based on the following peptide qualities:

- molecular weight of peptide

- hydrophobicity of peptide

- extent of predicted disorder of peptide

- low isoelectric point vs high isoelectric point

Sensitivity

N/A

Publication bias

Publication bias will be assessed using funnel plots.

Contact details for further information

Valerie Booth
vbooth@mun.ca

Organisational affiliation of the review

Memorial University of Newfoundland
https://www.mun.ca/biochem/

Review team members and their organisational affiliations

Ashan Wijesinghe. Memorial University of Newfoundland
Sarika Kumari. Memorial University of Newfoundland
Dr Valerie Booth. Memorial University

Review type

Pre-clinical animal intervention review

Anticipated or actual start date

01 September 2020

Anticipated completion date

17 March 2021

Funding sources/sponsors

Natural Sciences and Engineering Council of Canada Discover Grant to Valerie Booth

Grant number(s)

(RGPIN 05154)

Conflicts of interest

None known

Language

(there is not an English language summary)

Country

Canada

Stage of review
Review Ongoing

Subject index terms status
Subject indexing assigned by CRD

Subject index terms
Animals; Peptides

Date of registration in PROSPERO
09 December 2020

Date of first submission
27 November 2020

Stage of review at time of this submission

**Stage**	**Started**	**Completed**
Preliminary searches	Yes	Yes
Piloting of the study selection process	Yes	Yes
Formal screening of search results against eligibility criteria	Yes	Yes
Data extraction	Yes	Yes
Risk of bias (quality) assessment	Yes	Yes
Data analysis	Yes	Yes

Revision note
A few minor changes have been done to reviewer details and eligibility criteria (qualifying already specified criteria).

The record owner confirms that the information they have supplied for this submission is accurate and complete and they understand that deliberate provision of inaccurate information or omission of data may be construed as scientific misconduct.
The record owner confirms that they will update the status of the review when it is completed and will add publication details in due course.

Versions
09 December 2020
14 December 2020
07 May 2021
