# Supplementary material for: Conjugates for use in peptide therapeutics: A systematic review and meta-analysis
Source: PLoS One. 2022 Mar 8;17(3):e0255753. doi: 10.1371/journal.pone.0255753 (PMC8903268; doi:10.1371/journal.pone.0255753)
Supplement: Attachment — Submitted filename: Response to reviewers.docx [file pone.0255753.s007.docx]

We would like to thank the editor and the reviewers for the time and thought put into reviewing our manuscript. The work has been revised in response to the comments.

We have left the reviewer’s comments in black font and have added our point-by-point response in blue.

Reviewer #1: This systematic review and meta-analysis examined the available literature (2015-2020) and assessed it objectively to identify which biological and synthetic conjugates produce the largest improvement in therapeutic peptide half-life. Their search has been done in 3 databases, a total of 16 articles were included in the review and 5 in the meta-analysis part. This article appears to be appropriate for publishing, but there are a few problems that need to be corrected.
1. On page 5 the search date is until December 15, 2020, but on page 6 it is September 1, 2020.
2. In Prisma chart, included studies should be written 16, not 19.

Apologies for the confusion with dates. The date on page 5 (11 December 2020), was the date that we last searched the three databases to extract studies relevant to our review. It is a PRISMA requirement that the date that each database was last searched be specified – so this information was included. The date range mentioned on page 6 (1 Sept 2015 to 1 Sept 2020) is what we used to restrict the search to capture only studies published/made available online in last 5 years (rationalized in the paper). The paragraph in page 5 was adjusted as follows:

“…Where possible, the search was also limited by publication language (English) and date range (1^st^ Sept 2015 to 1^st^ Sept 2020) to reflect the characteristics highlighted in the eligibility criteria (Table 2). …The databases were last searched on 11 Dec 2020. “

The PRISMA flowchart has been adjusted accordingly.

Reviewer #2: Based on the requirements that PLOS ONE has for meta-analysis and systematic review publications, your paper does not mention the grant that was listed in the financial disclosure. PLOS ONE requires that any funding of the research be mentioned in the publication, as well as the role of the funders if they have one, as noted in their PRISMA 2009 Checklist for systematic reviews and meta-analysis submission guidelines.

The grant specified in the financial disclosure and the role of the funders has been added to the acknowledgements section of the manuscript as follows:

“This work was supported by a Discovery Grant from the Natural Sciences and Engineering Research Council (NSERC) of Canada to Valerie Booth (RGPIN 05154). The funding source was not involved in study design; in the collection, analysis and interpretation of data; in the writing of the report; or in the decision to submit the article for publication.”
